# Influence of Soldiers’ Cardiorespiratory Fitness on Physiological Responses and Dropouts During a Loaded Long-distance March

**DOI:** 10.1093/milmed/usab540

**Published:** 2022-01-07

**Authors:** Regina Oeschger, Lilian Roos, Thomas Wyss, Mark J Buller, Bertil J Veenstra, Rahel Gilgen-Ammann

**Affiliations:** Swiss Federal Institute of Sport Magglingen SFISM, Magglingen CH-2532, Switzerland; Swiss Federal Institute of Sport Magglingen SFISM, Magglingen CH-2532, Switzerland; Swiss Federal Institute of Sport Magglingen SFISM, Magglingen CH-2532, Switzerland; U.S. Army Research Institute of Environmental Medicine, Natick, MA 01760, USA; Institute of Training Medicine & Training Physiology, Royal Netherlands Army, Utrecht 3584 AB, The Netherlands; Swiss Federal Institute of Sport Magglingen SFISM, Magglingen CH-2532, Switzerland

## Abstract

**Introduction:**

In military service, marching is an important, common, and physically demanding task. Minimizing dropouts, maintaining operational readiness during the march, and achieving a fast recovery are desirable because the soldiers have to be ready for duty, sometimes shortly after an exhausting task. The present field study investigated the influence of the soldiers’ cardiorespiratory fitness on physiological responses during a long-lasting and challenging 34 km march.

**Materials and Methods:**

Heart rate (HR), body core temperature (BCT), total energy expenditure (TEE), energy intake, motivation, and pain sensation were investigated in 44 soldiers (20.3 ± 1.3 years, 178.5 ± 7.0 cm, 74.8 ± 9.8 kg, body mass index: 23.4 ± 2.7 kg × m^−2^, peak oxygen uptake ($\dot{\rm{V}}$O_2peak_): 54.2 ± 7.9 mL × kg^−1^ × min^−1^) during almost 8 hours of marching. All soldiers were equipped with a portable electrocardiogram to record HR and an accelerometer on the hip, all swallowed a telemetry pill to record BCT, and all filled out a pre- and post-march questionnaire. The influence of aerobic capacity on the physiological responses during the march was examined by dividing the soldiers into three fitness groups according to their $\dot{\rm{V}}$O_2peak_.

**Results:**

The group with the lowest aerobic capacity ($\dot{\rm{V}}$O_2peak_: 44.9 ± 4.8 mL × kg^−1^ × min^−1^) compared to the group with the highest aerobic capacity ($\dot{\rm{V}}$O_2peak_: 61.7 ± 2.2 mL × kg^−1^ × min^−1^) showed a significantly higher (*P* < .05) mean HR (133 ± 9 bpm and 125 ± 8 bpm, respectively) as well as peak BCT (38.6 ± 0.3 and 38.4 ± 0.2 °C, respectively) during the march. In terms of recovery ability during the break, no significant differences could be identified between the three groups in either HR or BCT. The energy deficit during the march was remarkably high, as the soldiers could only replace 22%, 26%, and 36% of the total energy expenditure in the lower, middle, and higher fitness group, respectively. The cardiorespiratory fittest soldiers showed a significantly higher motivation to perform when compared to the least cardiorespiratory fit soldiers (*P* = .002; scale from 1 [not at all] to 10 [extremely]; scale difference of 2.3). A total of nine soldiers (16%) had to end marching early: four soldiers (21%) in the group with the lowest aerobic capacity, five (28%) in the middle group, and none in the highest group.

**Conclusion:**

Soldiers with a high $\dot{\rm{V}}$O_2peak_ showed a lower mean HR and peak BCT throughout the long-distance march, as well as higher performance motivation, no dropouts, and lower energy deficit. All soldiers showed an enormous energy deficit; therefore, corresponding nutritional strategies are recommended.

## INTRODUCTION

In military service, marching is an important and very common task.^1^ The extensive distances covered on foot explain a relevant part of the greater physical demands on soldiers during military service compared to civilian life.^1^ This demanding workload results in high numbers of overuse injuries and therefore, a limited duty time.^2,3^ Previous studies have shown that a certain level of physical fitness protects soldiers from injuries.^4^ Moreover, Roos et al.^5^ showed that the aerobic performance of the soldier could predict musculoskeletal injury risk. Intervention studies conducted on this topic have revealed further approaches to minimize the frequency and severity of injuries. On the one hand, adapted marching with progressive increases in distance decreased the number of injuries and dropouts from the military service, whereas, on the other hand, training approaches that replaced long-distance runs with individualized high-intensity interval runs improved the aerobic performance of soldiers.^5,6^ Although progressively increasing marching distances and improved aerobic performance can reduce the risk of injuries during basic military training (BMT), little is known about the physical and mental conditions of soldiers during prolonged marching, which is an inevitable component of basic military training. Measurements of body core temperature (BCT) and heart rate (HR) become increasingly focused by research groups because it is recognized that incidents such as exertional heat illness can be detected during tasks like loaded marching.^7^

Endurance training induces numerous physiological changes in the body. Heat rate increases with increasing physical activity intensity, so these adaptations result in a lower HR at a given workload in endurance-trained individuals compared to untrained individuals.^8^ Another parameter that distinguishes between different training statuses of people is the HR recovery after exercise cessation.^8,9^ When an exercise is stopped, the body enters a state of rest by immediately reactivating the parasympathetic nervous system, which decreases the HR.^8,10^ In a second phase (the slow phase), the withdrawal of the sympathetic system plays an important role in HR recovery.^10^ Previous studies showed a faster HR recovery 30 to 120 seconds after exercise in trained individuals compared to untrained individuals.^9^ What also characterizes the endurance trained state is an increased skin blood flow at a given BCT.^11^ Skin blood flow has a positive effect on controlling BCT during exercise by bringing the blood close to the skin surface to allow heat dissipation from the body.^11,12^

These physiological adaptations attributable to high aerobic capacity are also expected to occur in soldiers throughout a long-distance march. Therefore, the aim of this study was to investigate the influence of the soldiers’ $\dot{\rm{V}}$O_2peak_ on HR, BCT, and recovery abilities during a loaded 34 km distance field march. Measurements of the energy deficit, motivation, pain sensation, and dropout related to the march were further aims. We hypothesized that soldiers with a higher cardiorespiratory fitness ($\dot{\rm{V}}$O_2peak_) will show a lower HR and BCT throughout a prolonged march and a faster recovery during the recovery breaks compared to their peers with lower aerobic capacity.^8,9,11^

## METHODS

### Subjects

Rescue soldiers of two companies of the Swiss Armed Forces who were undergoing 18 weeks of BMT for missions in disaster relief were asked to participate voluntarily in the study. The soldiers received oral and written information about the study design and provided written informed consent. The study was approved by the Cantonal Ethics Committee Bern, Switzerland (KEK-Nr. 082/15).

From total 230 informed soldiers, 67 agreed to take part in the study of which again 12 soldiers were already excluded before the march started for the following reasons: left the company (*n* = 1), completed no progressive endurance run (*n* = 1), assignment to another task instead of the march (*n* = 6), or dispensation for the march (*n* n = 4). In total, 55 male rescue soldiers started the march. The data of 44 rescue soldiers was finally used for analysis (Table I): 11 (20%) soldiers were excluded either because of no HR and BCT data due to technical problems (*n* = 2), or because they ended marching early (*n* = 8), or both (*n* = 1). Soldiers who dropped out were recorded but not included in the data analysis because the incomplete data sets could not be compared with the complete ones.

**TABLE I.** Characteristics of the Three Investigated Groups of Soldiers

	M ± SD [range: min, max]			
	Overall (*n *= 44)	LT (*n *= 15)	MT (*n *= 12)	HT (*n *= 17)
Age (years)	20.3 ± 1.3 [19.0, 24.0]	20.7 ±1.1 [19.0, 23.0]	19.8 ±1.1 [19.0, 22.0]	20.4 ±1.4 [19.0, 24.0]
Height (cm)	178.5 ± 7.0 [164.0, 193.0]	178.1 ± 6.5 [170.1, 191.9]	178.7 ± 7.3 [164.0, 187.1]	178.8 ± 7.5 [168.7, 193.0]
Weight (kg)	74.8 ± 9.8 [57.7, 110.0]	77.0 ± 12.5 [60.4, 110.0]	74.1 ± 9.6 [57.7, 93.6]	73.3 ± 7.2 [62.9, 90.1]
BMI (kg × m^−2^)	23.4 ± 2.7 [19.1, 33.6]	24.3 ± 3.9 [19.1, 33.6]	23.1 ± 2.0 [20.2, 26.7]	22.9 ± 1.6 [19.5, 26.0]
$\dot{\rm{V}}$ O_2peak_ (ml × kg^−1^ × min^−1^)	54.2 ± 7.9 [30.3, 65.8]	44.9 ± 4.8 [30.3, 50.3]	55.1 ± 2.1 [51.5, 57.9]	61.7 ± 2.2 [58.8, 65.8]

Abbreviations: M: mean; SD: standard deviation; min, max: range of variability; LT: lower third of cardiorespiratory fitness ($\dot{\rm{V}}$O_2peak_); MT: middle third of $\dot{\rm{V}}$O_2peak_; HT: higher third of $\dot{\rm{V}}$O_2peak_; BMI: body mass index.

### Experimental Protocol

One week before this study, all soldiers performed a mandatory progressive endurance run during their normal military routine.^13^ The progressive endurance run was accomplished outdoors on asphalt in sports clothes and shoes and started with a speed of 8.5 km × h^−1^ and increased by 0.5 km × h^−1^ every 200 m until voluntary exhaustion. These data were used to calculate $\dot{\rm{V}}$O_2peak._^13^$\dot{\rm{V}}$O_2peak_ is defined as the highest $\dot{\rm{V}}$O_2_ value attained during a particular test, whereas $\dot{\rm{V}}$O_2max_ defines the highest value attainable by a subject.^14^

The 34 km march took place at the end of the survival exercise week of the BMT in training week 17 in March in Switzerland. The scheduled start time was 3:00 pm. Eight hours before the march, every soldier swallowed an ingestible telemetry pill (Vitalsense, Mini Mitter Company, Bend OR, USA) as a valid measurement of BCT during exercise.^15^ Three hours prior the start, the body height and weight (Stadiometer: model 213; calibrated scale: model 861; Seca GmbH, Hamburg, Germany), as well as the additional load of clothing and packing (military clothing and packing order CNM 828: camouflage dress, jacket, headgear, boots, underwear [t-shirt and/or tricot shirt and/or fleece-jacket], 5.7 ± 1.1 kg; and backpack, basic carrying unit, double-bag, helmet and assault rifle, 17.6 ± 0.9 kg) were measured. The soldiers also filled out a pre-march questionnaire consisting of questions about any pain or physical complaints and their motivation to perform the march (scale from 1-10; 1 = not at all, 10 = extremely). Each soldier was fitted with a sensor belt around his chest that included a two-lead electrocardiogram and a sensor electronics module (SEM; Equivital™ EQ-02, Hidalgo Ltd., Cambridge, UK) that stored information on HR and the continuous wireless signal of the soldier’s telemetry pill. Each solider was also equipped with an accelerometer (H-Acc; PARTwear, HuCE microLab, Biel, Switzerland) worn in a belt pouch on the hip. Seven randomly selected soldiers were also fitted with a global positioning system (GPS) device (V800; Polar Electro Oy, Kempele, Finland) that recorded marching distance, speed, and time. After these preparations, just before the start of the march, the soldiers rested in a supine position for at least 15 minutes to measure resting HR.^16^

The task of the soldiers was to march as a squad at all times and to reach the finish together. During the march, the soldiers had four recovery breaks. Eating and drinking occurred ad libitum during these breaks. Directly after the march, the level of pain (scale from 1-10; 1 = not at all, 10 = extremely) and the eating behavior (what and how much) were documented with a post-march questionnaire. The caloric values of the consumed food items were obtained from the package label or were calculated with the online software of Imkenberg and Mauch.^17^

### Data Processing and Statistics

The individual $\dot{\rm{V}}$O_2peak_ of the 55 soldiers who performed the march was estimated using the following regression: $\dot{\rm{V}}$O_2peak_ (mL × kg^−1^ × min^−1^) = 2.309 × velocity_peak_ (km × h^−1^) + 16.549.^13^ Thereafter, the soldiers were divided into three fitness groups according to their $\dot{\rm{V}}$O_2peak_ values, with mathematical allocation to the lower third (LT), the middle third (MT), and the higher third (HT).

All HR, BCT, and H-Acc data were processed in 1-minute intervals. The second phase of the recovery ability was analyzed using the HR and BCT data obtained during the third break of the march. In detail, the computations included the soldiers’ HR and BCT at the last minute before the break (HR_break,-1_ and BCT_break,-1_) and at the tenth minute of the break (HR_break,10_ and BCT_break,10_). The start of the break was defined as the minute at which the step frequency (computed with the SEM) dropped below 1 Hz. The difference between the values BCT_break,-1_ and BCT_break,10_ was documented as BCT recovery. The same strategy was applied for HR recovery ([HR_break,-1_]_—_[HR_break,10_]).

The energy deficit was calculated as energy intake minus total energy expenditure (TEE) of the march, where TEE was the sum of the physical activity energy expenditure (PAEE) and resting energy expenditure (REE). Resting energy expenditure was calculated using anthropometric data and the formula for men by Mifflin et al.^18^ while PAEE was calculated based on HR and H-Acc and the formulas according to Wyss and Mader.^16^ A step frequency greater than 1 Hz was defined as marching with a backpack (PAEE (kcal × min^−1^) = [HR above the resting HR (HRaR; beats × min^−1^)  ×0.7810 − 15.9337]/4.1868), whereas a step frequency lower than 1 Hz was defined as a break (PAEE (kcal × min^−1^) = [HRaR (beats × min^−1^)  ×0.4840 + H-Acc (cpm)  ×  0.0010 − 4.7964]/4.1868).^16,19^

Data were tested for normal distribution with the Shapiro–Wilk test. Differences between the three fitness groups were analyzed by one-way analysis of variance (ANOVA) and Bonferroni *post hoc* tests and the group results were documented as mean ± standard deviation as well as ranges (minimal and maximal values). Ordinally scaled data (motivation and pain sensation) or not normally distributed data (PAEE, TEE, solid food intake, and energy deficit) were tested with the Kruskal–Wallis Test and Dunn–Bonferroni *post-hoc*. In these cases, group results were presented as median (Mdn) and inter quartile range (IQR). The frequency of dropouts was also tested using the Fisher exact test. An α-level of *P* < .05 was set as the level of significance for all tests.

## RESULTS

**FIGURE 1.**
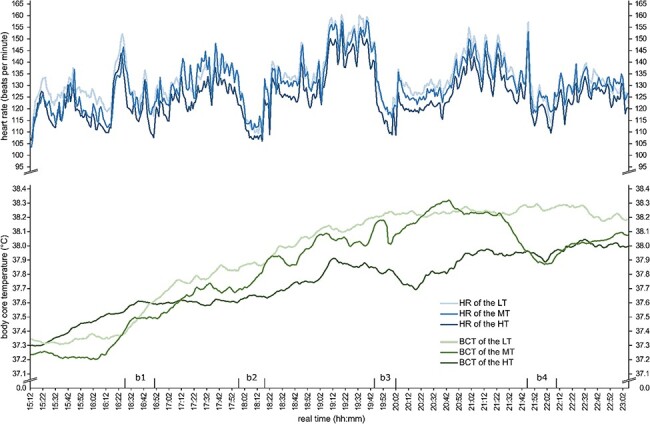
Continuous data for heart rate (HR) and body core temperature (BCT) during a loaded 34-km march by Swiss Armed Forces rescue soldiers divided into three groups according to cardiorespiratory fitness. LT: lower third of cardiorespiratory fitness ($\dot{\rm{V}}$O_2peak_); MT: middle third of $\dot{\rm{V}}$O_2peak_; HT: higher third of $\dot{\rm{V}}$O_2peak_; b: break.

The total measured marching distance according to the Global Positioning System (GPS) devices was 33.64 ± 0.04 km, with a mean marching speed (break periods excluded) of 4.83 ± 0.84 km × h^−1^. The starting point was 565 m above sea level and the soldiers hat to overcome 772 m negative altitude and 467 m positive altitude. The march duration was 7:55 hours (from 3:11 pm until 11:06 pm) for all soldiers, with four breaks lasting between 19 and 24 minutes. The changes of the meteorological data from the beginning to the end of the march are presented in Table II. The age, height, weight, and body mass index of the 44 soldiers did not differ significantly between the LT, the MT, and the HT (Table I). The mean HR was significantly different between the groups (*P = *.039), and post hoc analysis (Bonferroni) showed that, throughout the march, the soldiers in the HT had a significantly lower HR compared to soldiers grouped into the LT (Table III, Figure 1). The three fitness groups showed no significant differences in peak HR, mean BCT, or peak BCT (*P = *.050); however, the subsequent post hoc analysis revealed a significantly higher peak BCT in the LT compared to the HT (Table III). In terms of recovery ability during the break, the three groups showed no significant differences in either the HR or the BCT decreases.

**TABLE II.** Meteorological Data during the 34-Km March

			Daytime (hh:mm)								
	Min	Max	15:10	16:10	17:10	18:10	19:10	20:10	21:10	22:10	23:10
Air temperature (°C)	2.1	6.6	6.4	6.4	5.2	4.4	3.8	3.4	2.4	2.3	2.1
Air pressure (hPa)	931	935	931	932	932	933	934	934	934	935	935
Wind speed (km × h^−1^)	6.5	19.4	9.4	10.4	11.9	14.4	17.6	15.8	6.8	10.1	14.0
Humidity (%)	52	86	56	54	60	66	74	76	82	85	84

**TABLE III.** Heart Rate, Body Core Temperature, Energy Intake and Energy Expenditure of the Three Investigated Groups

	M ± SD [range: min, max]					
	Overall (*n *= 44)	LT (*n *= 15)	MT (*n *= 12)	HT (*n *= 17)	*F*-value	*post-hoc*
HR mean (bpm)	129 ± 9 [109, 156]	133 ± 9 [119, 156]	130 ± 8 [115, 146]	125 ± 8 [109, 141]	3.52*	LT> HT*
HR peak (bpm)	165 ± 8 [147, 188]	168 ± 9 [155, 188]	167 ± 6 [156, 176]	162 ± 8 [147, 181]	2.74	
HR recovery (bpm)	28 ± 8 [11, 52]	30 ± 8 [15, 52]	28 ± 8 [12, 41]	26 ± 8 [11, 37]	0.97	
BCT start (°C)	37.3 ± 0.2 [36.8, 37.8]	37.3 ± 0.2 [36.8, 37.7]	37.2 ± 0.2 [36.8, 37.5]	37.3 ± 0.2 [36.9, 37.8]	0.73	
BCT mean (°C)	37.8 ± 0.3 [37.3, 38.5]	37.9 ± 0.2 [37.6, 38.3]	37.8 ± 0.3 [37.4, 38.3]	37.7 ± 0.3 [37.3, 38.5]	2.98	
BCT peak(°C)	38.5 ± 0.3 [38.1, 39.1]	38.6 ± 0.3 [38.1, 39.1]	38.5 ± 0.3 [38.2, 38.9]	38.4 ± 0.2 [38.1, 39.0]	3.22	LT> HT*
BCT recovery (°C)	0.0 ± 0.1 [−0.3, 0.3]	0.0 ± 0.1 [−0.2, 0.2]	0.0 ± 0.2 [−0.3, 0.3]	0.0 ± 0.1 [−0.2, 0.2]	1.13	
	Mdn; IQR [range: min, max]				Kruskal–Wallis H	
TEE (kcal)	4,773; 780 [3,153, 7,008]	4,803; 962 [3,942, 7,008]	4,684; 430 [4,302, 6,321]	4,732; 1,189 [3,153, 5,919]	0.26	
PAEE (kcal)	4,177; 721 [2,625, 6,391]	4,235; 903 [3,335, 6,391]	4,077; 388 [3,732, 5,747]	4,136; 1,162 [2,625, 5,370]	0.28	
Solid food intake (kcal)	1,250; 1,020 [234, 2,857]	1,042; 631 [512, 2,647]	1,212; 561 [234, 2,752]	1,718; 887 [608, 2,857]	5.12	
Energy deficit (kcal)	−3,614; 1,317 [−6,087, −1,517]	−3,848; 454 [−5,966, −1,660]	−3,614; 1,160 [−6,087, −1,550]	−3,047; 1,277 [−4,219, −1,517]	5.59	

* *P* < 0.05, critical F-value = 3.23, critical H-value = 5.99; Abbreviations: M: mean; SD: standard deviation; min, max: range of variability; LT: lower third of cardiorespiratory fitness ($\dot{\rm{V}}$O_2peak_); MT: middle third of $\dot{\rm{V}}$O2peak; HT: higher third of $\dot{\rm{V}}$O_2peak_; HR: heart rate; bpm: beats per minute; BCT: body core temperature; °C: degree Celsius; Mdn: median; IQR: interquartile range; TEE: total energy expenditure; PAEE: physical activity energy expenditure; kcal: kilocalories.

Comparison of the TEE throughout the march revealed no significant differences in the intake of solid food during the recovery phases of the march or in the energy deficit between the groups (Table III). Remarkably, the energy deficit during the march was high, as the groups could only replace LT = 22%, MT = 26%, and HT = 36% of TEE.

The Kruskal–Wallis test showed differences in motivation before the march in the three fitness groups (Mdn = 6.0, IQR = 3.0, *P* = .003), with a significantly higher motivation before the march in the HT (Mdn = 7.3, IQR = 2.0) than in the LT (Mdn = 5.0, IQR = 2.0, *P* = .002). Comparison of the outcomes of pain pre-march (Mdn = 2.0, IQR = 1.0) and post-march (Mdn = 7.0, IQR = 2.8) revealed no significant difference between the three groups.

Nine soldiers (16%) had to end marching early: four soldiers (21%) from the LT and five from the MT (28%), but none from the HT. The frequency of dropout of the soldiers was dependent on the tertile (Fisher’s exact test = 6.1, *P* = .049), with a significant difference observed in the proportion between the MT and the HT (*P* = 0.045). Additionally, the pain pre-march values and the dropout rate were significantly correlated (Fisher’s exact test = 24.4, *P* < 0.001), with higher pre-march pain values demonstrated in the soldiers who had to end marching early. Moreover, 18 soldiers reported a pre-march musculoskeletal pain sensation in a specific body region. Of the nine soldiers who had to end marching early, eight reported musculoskeletal pain already pre-march, in three soldiers the complaints reported before and after the march corresponded. In total, the following complaints or painful body regions (number of indications) were reported post-march by the soldiers who had to end marching early: Achilles tendon (2), foot (1), knee (1), back (1), muscular cramps (1), blisters (1), blisters and muscular cramps and knee pain (1), and no indication (1).

## DISCUSSION

The soldiers in the present study were all marching at the same speed and the same additional load. However, since the $\dot{\rm{V}}$O_2peak_ differed greatly between the three groups, the respective soldiers marched at different relative $\dot{\rm{V}}$O_2peak_ values. This impact is not only visible in the HR as well as in the BCT data, but also in the ability of food intake. Ideally, soldiers with lower $\dot{\rm{V}}$O_2peak_ values would have to march at a lower absolute speed to successfully complete the marching task.^20,21^

The HR results were consistent with the expected behavior of HR under similar submaximal exercise intensity in soldiers who differ in aerobic capacity.^8^ A lower HR at the same absolute work intensity results in a reduced relative task intensity, thereby allowing longer task performance and/or greater capacity for repeated efforts. However, the overall mean HR (129 ± 9 bpm) was higher than the value reported by Pihlainen et al.^22^ who measured a mean HR of 123 ± 9 bpm in a loaded march of soldiers of the Finnish Army (mean $\dot{\rm{V}}$O_2max_ of 48.1 ± 4.4 mL × kg^−1^ × min^−1^). Notably, in that study, the Finnish Army marched only 13.31 km in 3:13 hours and had three breaks and a walking speed of 5.4 km × h^−1^. Despite the lower $\dot{\rm{V}}$O_2max_ values compared to the MT and HT and the higher walking speed, the soldiers of the Finnish Army had a lower mean HR compared to all our fitness groups. Although the additional load was comparable (24.4 kg in the Finnish to 23.2 kg in the Swiss study), comparison of the HR in different settings seems almost impossible, as the total distance, marching time, terrain, and weather conditions, as well as the number and duration of breaks, also influence HR values.

The BCT at the beginning of the march and the mean BCT were the same in all three groups, and yet, during the march, the peak BCT was significantly higher in the LT than in the HT. Therefore, we confirmed the previous findings that aerobic capacity influences BCT.^11,12^ However, this contrasted with the findings of Jay et al.^23^ who presented BCT data obtained on a cycle ergometer in a laboratory setting. They concluded that groups with different aerobic capacities ($\dot{\rm{V}}$O_2peak_ of 40.3 ± 2.9 and 60.1 ± 4.5 mL × kg^−1^ × min^−1^) showed a lower BCT at the end of an exercise due only to a lower BCT at the beginning of the exercise and not due to differences in $\dot{\rm{V}}$O_2peak_. Interestingly, a visual inspection revealed that the mean BCT of the MT showed a delayed increase at the beginning and a decrease in the BCT during the last recovery break, which we cannot yet explain.

The ability to maintain operational readiness and the associated resilience, as well as the ability to recover within a short time after physical stress, is becoming increasingly important in the military context.^24^ In the present study, neither the HR nor the BCT showed differences in the recovery ability in all three fitness groups. In fact, the HR recovered to the same extent in all groups, whereas the BCT did not decrease during the first ten minutes of the third recovery break in any group.

Comparison of the PAEE with Howley’s table of occupational work intensity indicated that this march, for all soldiers, was a “very heavy” task (>31.4 kJ  ×  min^−1^ or >7.5 kcal  ×  min^−1^).^25^ In addition, with an overall energy deficit of about 3,600 kcal, the TEE was far from compensated by the food consumption. Such considerably negative energy balances were also observed by Stenberg^26^ during extensive military tasks.

Although the results were not significantly different, the LT and MT tended to show a greater energy deficit compared to the HT. During the march, the LT and the MT were able to refuel 22% and 26% of TEE, respectively, while the HT could replace 36% of the TEE. Temporary appetite suppression is a known post-exercise response and is likely to occur during the short recovery breaks of a march, meaning that the soldiers may not consume much food.^27–29^ This appetite suppression may also depend on the intensity of the exercise.^27^ More intense exercise is associated with greater suppression, whereas the fitness level of the soldiers have an influence on appetite regulation.^27^ Consequently, a higher $\dot{\rm{V}}$O_2peak_ results in a lower relative exercise intensity and less appetite suppression, leading to a lower energy deficit in the HT compared to the LT and the MT. Therefore, the nutritional recommendations, such as the composition and amounts of macronutrients during loaded marching events, need further investigation.^30^

The findings documented by Costa et al.^31^ together with the energy deficit detected in our soldiers, indicate that full replacement of EE during this type of march is highly unlikely. Hence, rather than focusing on the entire energy requirement to be covered during the march, more attention is needed on consumption of what is feasible and also tolerable, because appetite disorders or gastrointestinal symptoms are common during endurance exercises, although perhaps less pronounced at lower exercise intensities.^31^ Consequently, nutritional strategies aimed at reducing the extreme energy deficit are strongly recommended. In particular, the meals before and in the hours or days following a long distance march may play a major role. Operational readiness is a key requirement for soldiers; therefore, regeneration of energy stores and induction of the refueling process need to occur as rapidly and effectively as possible. In addition to physical performance, adequate nutrition contributes to a healthy and resilient soldier, trough the positive impacts on injury prevention, recovery time, immune system function, well-being, and maintaining troop morale.^32^

In addition to showing a higher performance motivation, the soldiers with the highest cardiorespiratory fitness were also less likely to end marching early, but a correlation was noted between already existing pain sensation before the march and subsequent dropout. Musculoskeletal injuries attributable to loaded road marching are one of the leading injury causes for soldiers’ limited military readiness.^3,33^ Unfortunately, the exact reasons for discontinuation were not recorded, but all soldiers that ended marching early indicated severe pain in a specific region of the body. While musculoskeletal pain and injuries may be reduced with improved physical performance, blisters are most likely attributable to footwear that is not optimally fitted.

## LIMITATIONS

One limitation of this study is that energy intake was only collected post-march and by questionnaire. Although these data may be flawed, the energy deficit was certainly still considerable.

Typically, soldiers are pre-fatigued at the end of the survival exercise week due to increased physical demands and reduced night rest of 6 hours per night. Possibly, this fact had also an impact on physiological parameters of the present study.

## CONCLUSIONS

This field study revealed a relationship between higher cardiorespiratory fitness and lower mean HR and peak BCT values throughout the long distance march. During the breaks, no evidence indicated a different recovery behavior for HR or BCT between the three groups. The rapid recovery of the energy stores should be given the utmost attention, as the entire sample showed an enormous energy deficit regardless of fitness level. A higher $\dot{\rm{V}}$O_2peak_ was also correlated with a higher motivation to perform and less likelihood of dropout from the march. Strategies to promote a higher $\dot{\rm{V}}$O_2peak_ (starting at 58.4 mL × kg^−1^ × min^−1^) and better nutrition are suggested to avoid restricted operational readiness due to pain or injury and to keep exercise recovery times as short as possible.
